# Data on genotypic distribution and linkage disequilibrium of several ANRIL polymorphisms in hemodialysis patients

**DOI:** 10.1016/j.dib.2017.02.011

**Published:** 2017-02-12

**Authors:** A. Arbiol-Roca, A. Padró-Miquel, M. Hueso, E. Navarro, P. Alía-Ramos, M.T. González-Álvarez, I. Rama, J. Torras, J.M. Grinyó, J.M. Cruzado, N. Lloberas

**Affiliations:** a Biochemistry Department, IDIBELL, Hospital Universitari de Bellvitge, Barcelona, Spain; b Nephrology Department, IDIBELL, Hospital Universitari de Bellvitge, Barcelona, Spain; c Molecular Oncology Laboratory, IDIBELL, Barcelona, Spain

**Keywords:** ANRIL, Polymorphisms, Linkage disequilibrium, Hemodialysis

## Abstract

A long non-coding RNA called ANRIL located on chromosome 9p21.3 has been identified as a novel genetic factor associated with cardiovascular disease. Investigation of several single nucleotide polymorphisms (SNPs) of Noncoding Antisense RNA in the INK4 Locus (ANRIL) gene are of particular interest. This article reports data related to the research article entitled: “Association of ANRIL gene polymorphisms with major adverse cardiovascular events in hemodialysis patients” (Arbiol-Roca et al. [1]). Data presented show the genotypic distribution of four selected ANRIL SNPs: rs10757278, rs4977574, rs10757274 and rs6475606 in a cohort constituted by 284 hemodialysis patients. This article analyzes the Hardy-Weinberg disequilibrium of each studied SNP, and the linkage disequilibrium between them.

**Specifications Table**

**Table:** 

Subject area	Genetics
More specific subject area	Molecular genetics, hemodialysis research
Type of data	Tables and figure
How data was acquired	7900 HT Fast Real-Time PCR System (Applied Biosystems (ThermoFisher, USA)), SDS 2.3 software (Applied Biosystems, ThermoFisher, USA), STATA 12.0 software and Haploview software (Broad Institute, USA)
Data format	Analyzed
Experimental factors	Genomic DNA was extracted from whole blood samples
Experimental features	Genotyping of four SNPs was carried out using real time PCR with TaqMan validated probes (Foster City, CA)
Data source location	Hospital Universitari de Bellvitge, Hospitalet de Llobregat, Barcelona, Spain
Data accessibility	The data is available within this article

**Value of the data**

• This dataset provides several ANRIL SNPs frequencies in an hemodialysis cohort of patients.
• The selected SNPs rs10757278, rs4977574, rs10757274 and rs6475606 follow the Hardy-Weinberg equilibrium and are in linkage disequilibrium.
• rs10757278 ANRIL SNP can be a representative SNP of a strong linkage disequilibrium block that showed significant genotypic association with major adverse cardiovascular events in patients on hemodialysis [1].
• Our data can provide some insight into ANRIL haplotype patterns.

1. **Data**

Table 1 describes genotypic frequencies regarding several ANRIL SNPs in an hemodialysis cohort of patients together with the corresponding Hardy-Weinberg p-values.

Table 2 summarizes the main statistical parameters in the linkage disequilibrium analysis and Fig. 1 illustrates the Haploview linkage disequilibrium plot.

## 2. Experimental design, materials and methods

### 2.1 Sample collection and genomic DNA extraction

This research was designed to be an observational follow-up study. Two hundred and eighty four chronic kidney patients that started on hemodialysis were collected from Bellvitge University Hospital. The study protocol was approved by the local Ethics Committee and informed written consent was obtained from all recruited subjects. Genomic DNA was extracted from peripheral blood of patients using the Wizard^®^ Genomic DNA Purification Kit (Promega Corporation, Sydney, Australia) and was stored at −80 °C until analysis

### 2.2 DNA genotyping

Genotyping of the SNPs (rs10757278, rs4977574, rs10757274 and rs6475606) was carried out with real-time PCR using TaqMan SNP Genotyping Assay (Applied Biosystems, Foster City, CA, USA) in 384-well plates that included positive and negative controls. TaqMan PCR and genotyping analyses were carried out on the 7900HT Fast Real-time PCR System, Applied Biosystems (Thermo Fisher Scientific), according to the manufacturer׳s instructions. The SDS 2.3 software (Applied Biosystems, ThermoFisher, USA) was used for allelic discrimination.

**Fig. 1:**
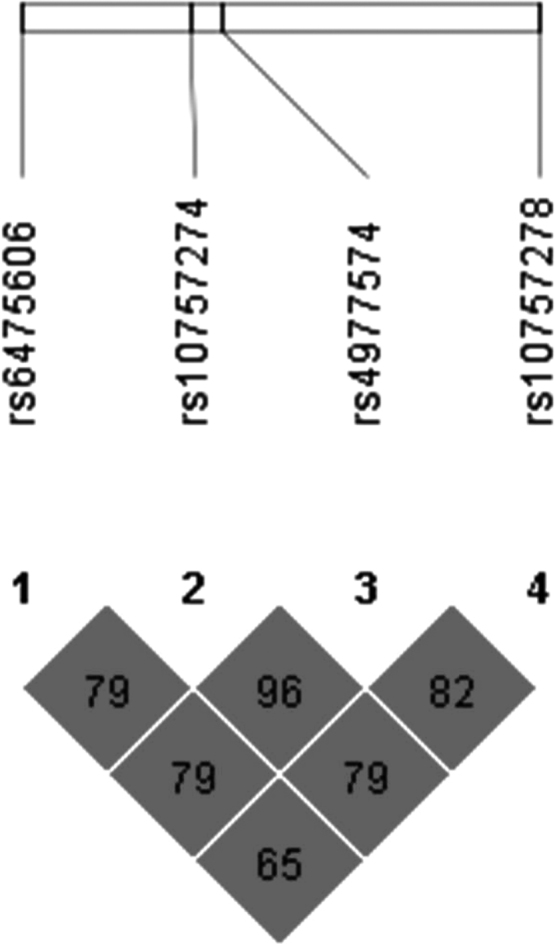
Linkage disequilibrium plot generated by Haploview software showing *r*^2^ values within diamonds for selected ANRIL SNPs examined in hemodialysis patients. Significant combined *P* values are highlighted in dark grey.

Allele frequency distribution was tested for Hardy-Weinberg equilibrium (Table 1) using *P* value of the Fisher׳s exact test:

Haploview software was used to verify the linkage disequilibrium pattern and for deducing the haplotype [2] (Table 2 and Fig. 1).

## Figures and Tables

**Table 1:** Genotype distributions of the polymorphisms (%) and *P* Hardy-Weinberg disequilibrium test.

			***P*****Hardy-Weinberg**
**rs10757278 A/G**			
AA	AG	GG	
23	53	24	0.64
**rs4977574 A/G**			
AA	AG	GG	
24	48	28	0.85
**rs10757274 A/G**			
AA	AG	GG	
24	49	27	0.98
**rs6475606 C/T**			
CC	CT	TT	
20	49	31	0.97

*P* value for Fisher׳s exact test (Hardy-Weinberg disequilibrium test).

**Table 2:** Linkage disequilibrium of investigated SNPs.

SNP1	SNP2	D’	r2	*P*
rs10757278	rs4977574	0,94	0,82	<0,0001
rs10757278	rs10757274	0,91	0,79	<0,0001
rs10757278	rs6475606	0,89	0,65	<0,0001
rs4977574	rs10757274	0,99	0,96	<0,0001
rs4977574	rs6475606	0,96	0,79	<0,0001
rs10757274	rs6475606	0,96	0,79	<0,0001

*SNP:* single nucleotide polymorphism*, D’* coefficient of linkage disequilibrium normalized, *r2* correlation coefficient, *P* value pairwise comparison test.
