# Supplementary material for: Data on genotypic distribution and linkage disequilibrium of several ANRIL polymorphisms in hemodialysis patients
Source: Data Brief. 2017 Feb 12;11:221–4. doi: 10.1016/j.dib.2017.02.011 (PMC5320056; doi:10.1016/j.dib.2017.02.011)
Supplement: Supplementary file 1 — Supplementary material [file mmc1.pdf]

Conflicts of Interest Statement DIB-D-16-00988

Manuscript title: DATA ON GENOTYPIC DISTRIBUTION AND LINKAGE DISEQUILIBRIUM OF SEVERAL ANRIL POLYMORPHISMS IN HEMODIALYSIS PATIENTS

The authors whose names are listed immediately below certify that they have NO affiliations with or involvement in any organization or entity with any financial interest (such as honoraria; educational grants; participation in speakers' bureaus; membership, employment, consultancies, stock ownership, or other equity interest; and expert testimony or patent-licensing arrangements), or non-financial interest (such as personal or professional relationships, affiliations, knowledge or beliefs) in the subject matter or materials discussed in this manuscript.

Author names:

Arbiol-Roca A, Padró-Miquel A, Hueso M, Navarro E, Alía-Ramos P, González-Álvarez MT, Rama I, Torras J, Grinyó JM, Cruzado JM and Lloberas N

The authors whose names are listed immediately below report the following details of affiliation or involvement in an organization or entity with a financial or non-financial interest in the subject matter or materials discussed in this manuscript. Please specify the nature of the conflict on a separate sheet of paper if the space below is inadequate.

Author names:

We confirm that there are no known conflicts of interest associated with this publication and there has been no significant support for this work that could have influenced its outcome.

We further confirm that manuscript has been approved by all named authors.

Arbiol-Roca A, Padró-Miquel A, Hueso M, Navarro E, Alía-Ramos P, González-Álvarez MT, Rama I, Torras J, Grinyó JM, Cruzado JM and Lloberas N

This statement is signed by all the authors to indicate agreement that the above information is true and correct (*a photocopy of this form may be used if there are more than 10 authors*):

Author's name (typed)	Author's signature	Date
Arbiol-Roca A		12-01-2017
Padró-Miquel A		12-01-2017
Hueso M		18-01-2017
Navarro E		12-1-2017
Alía-Ramos P		18/1/2017
González-Álvarez MT		12-1-2017
Rama I		12/1/2017
Torras J		18-1-2017
Grinyó JM		19-1-17
Cruzado JM		19-01-2017
Lloberas N		10/1/2017
